# Staged subtotal aortic replacement for an extensive aortic dissecting aneurysm in a 13-year-old girl with patent ductus arteriosus

**DOI:** 10.1016/j.xjtc.2023.04.004

**Published:** 2023-04-19

**Authors:** Kaito Omine, Shigeki Koizumi, Yosuke Inoue, Koki Yokawa, Kenta Masada, Yoshimasa Seike, Hiroaki Sasaki, Hitoshi Matsuda

**Affiliations:** Department of Cardiovascular Surgery, National Cerebral and Cardiovascular Center, Suita/Osaka, Japan

**Figure:**
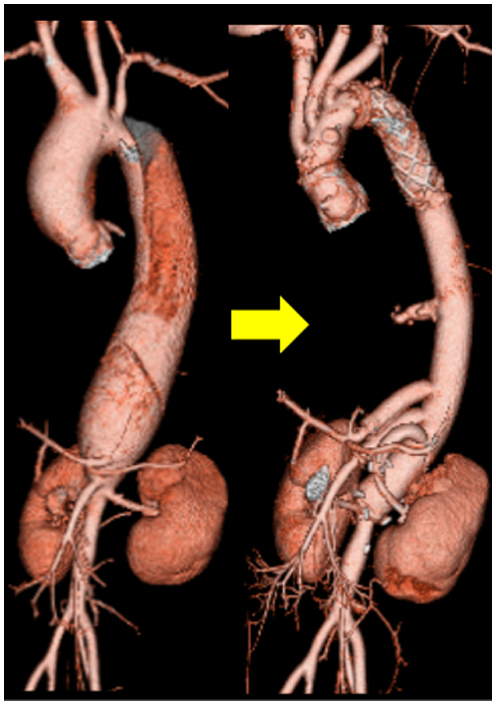
Preoperative and postoperative computed tomographic angiography.

**Central Message**

Subtotal aortic replacement was successfully completed using frozen elephant trunk for extensive aortic dissecting aneurysm with recanalized patent ductus arteriosus caused by *ACTA2* mutation.

Mutations in the actin, alpha-2, smooth muscle, aorta (*ACTA2*) gene is among the main causes of familial thoracic aortic aneurysms and dissections. Patent ductus arteriosus (PDA) in childhood or acute aortic dissection during teenage years has been reported as relatively frequent complication in patient with *ACTA2* mutation.^1^

In patient with *ACTA2* mutation with thoracoabdominal aortic aneurysm, PDA may complicate surgeries requiring cardiopulmonary bypass (CPB) because of left-to-right shunt and strong adhesion.^2^ Although several reports describe usefulness of frozen elephant trunk (FET) for closure of a PDA, use of FET for connective tissue disorder has been often reported negatively.^3^

Herein, we describe the successful treatment of a patient with an *ACTA2* mutation with recurrent PDA and acute type B aortic dissection complicated by thoracoabdominal aortic aneurysm.

This report was included in an investigational study approved by our institutional review board (M30-057; September 5, 2018). Written informed consent for publication was obtained from the patient.

## Case Report

A 13-year-old girl experiencing abrupt epigastric pain was referred to our hospital. She had previously undergone PDA ligation via median sternotomy followed by coil embolization for PDA recanalization before she was aged 1 year. However, recurrent recanalization was detected 6 months before the onset of this presentation.

**Figure 1:**
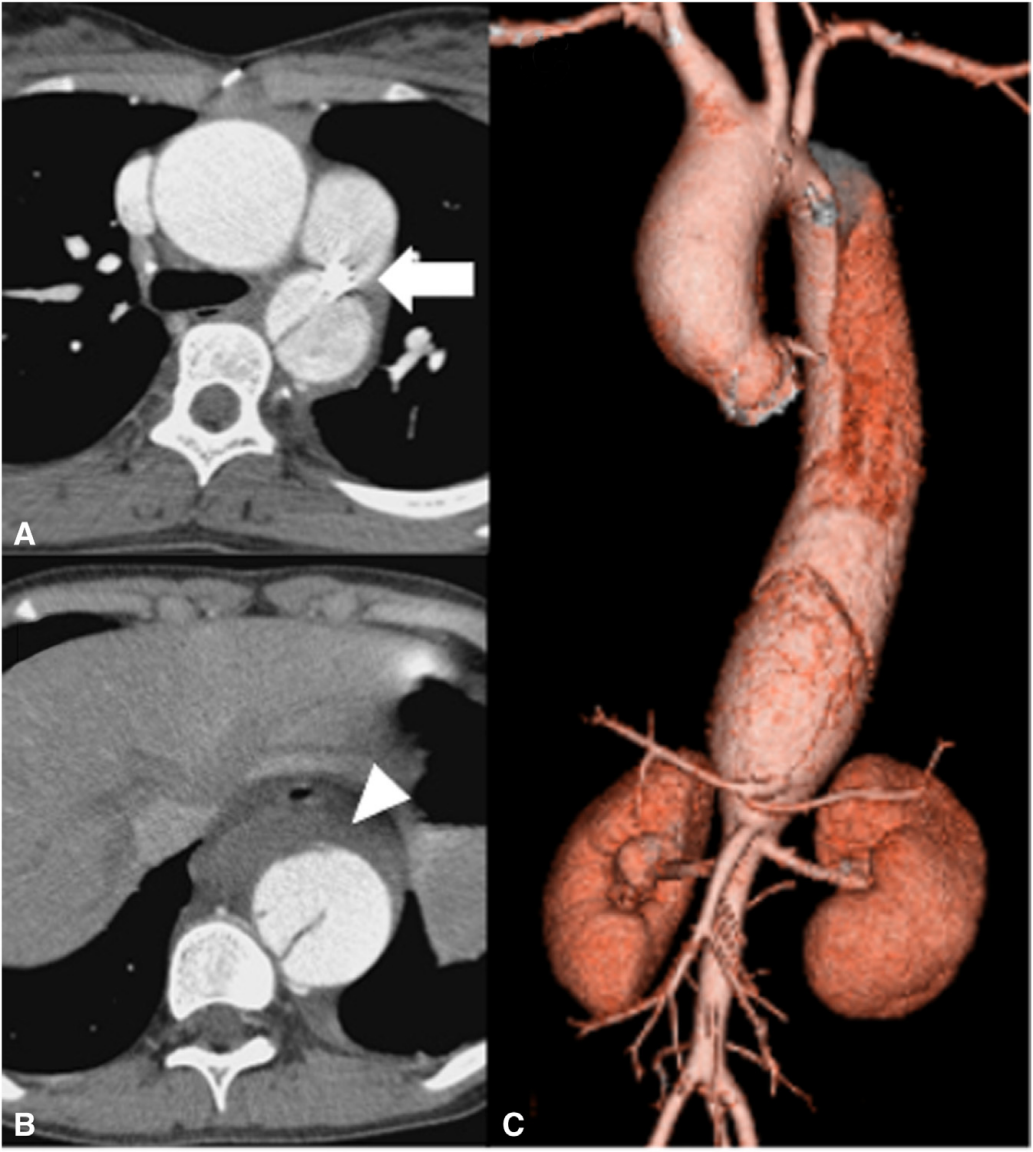
Computed tomography angiography on admission. A, Ascending aortic aneurysm (43 mm) and patent ductus arteriosus with the coil used for previous intervention (*arrow*). B, Type B aortic dissection with the entry at middle descending aorta surrounded by a low-density area (*arrowhead*). C, Three-dimensional computed tomography showing extensive aortic disease with ascending aortic aneurysm and type B aortic dissection.

Computed tomography angiography (CTA) revealed type B aortic dissection with a large primary entry at the descending aorta surrounded by a low-density area (Figure 1) and described no signs of visceral ischemia related to the dissection. Ascending aortic enlargement (43 mm) was concomitantly detected. Shrinkage of the low-density area was confirmed under optimal blood pressure control. Because strong adhesion around recurrent PDA was supposed, which was unsuitable for proximal arch clamp for partial bypass, staged complete aortic repair was planned.

On day 14 of admission, CPB was initiated with ascending aorta cannulation and bicaval drainage to perform total arch replacement (TAR). Under lower body circulatory arrest at 25 °C, the PDA was sutured using autopericardium inside the pulmonary trunk. The aortic arch was transected just distal to the left common carotid artery and an FET (21 × 90 mm) (J Graft Frozenix; Japan Lifeline) was inserted covering the PDA orifice inside the aorta. Thereafter, TAR was completed under selective antegrade cerebral perfusion using a 4-branched graft (22 mm Gelweave 4Branch; Terumo) (Video 1). On postoperative CTA, the PDA was effectively closed, and the false lumen of the proximal descending aorta surrounding the FET was thrombosed.

Five days after TAR, the patient was placed in a right semirecumbent position, and a left thoracotomy was created through the sixth intercostal space linking retroperitoneal laparotomy and a diaphragm incision. After femorofemoral partial CPB and active cooling was established at 30 °C, the FET was clamped at the Th6 level. A proximal stump was made at the edge of the FET, followed by a running suture with 4-branched graft (20 mm Gelweave Coselli Thoracoabdominal Graft; Terumo). Under visceral perfusion, the 11th intercostal artery, which was confirmed as the feeder of the Adamkiewicz artery on CTA, was reconstructed by the graft interposition technique. The aorta was beveled between the upper left and lower right renal arteries for distal anastomosis to match the prosthetic graft diameter, then the visceral arteries were individually reconstructed (Video 2). Preoperative cerebrospinal fluid drainage was not planned for fear of iatrogenic complications.

**Figure 2:**
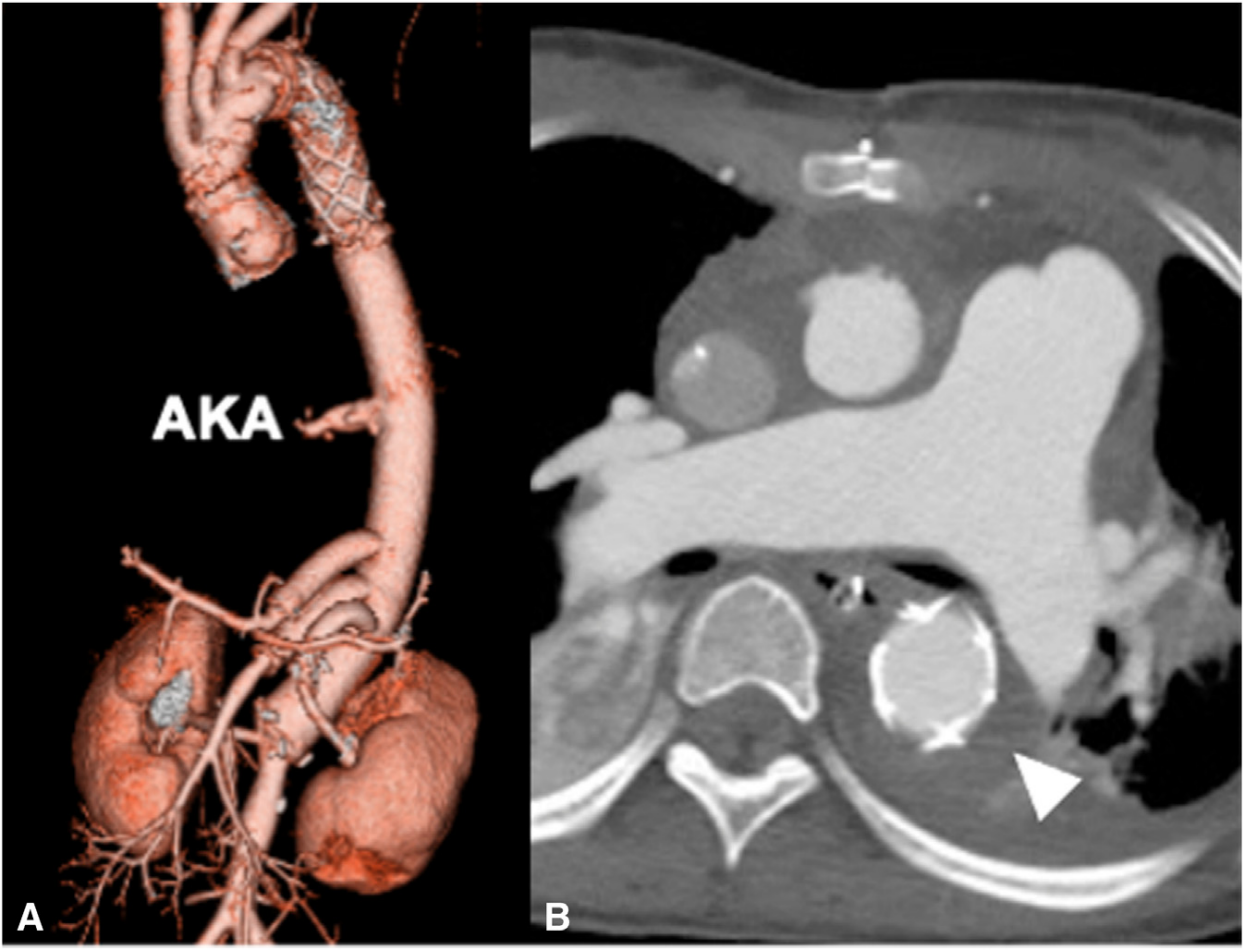
Postoperative computed tomography angiography. A, Successful staged extensive aortic replacement. Total arch replacement was performed using the frozen elephant trunk technique, which was anastomosed to a thoracoabdominal graft at the Th6 level. The Adamkiewicz artery (*AKA*) and reconstructed visceral branches were all open. B, False lumen around the frozen elephant trunk was thrombosed (*arrowhead*).

The day after the second surgery, she developed paraparesis but quickly recovered after spinal drainage and blood pressure augmentation. Postoperative CTA indicated aortic remodeling around the FET (Figure 2). Subsequently, she was discharged without any symptoms and later diagnosed with *ACTA2* mutation after a genetic examination.

## Discussion

Patients with *ACTA2* mutations tend to experience type B dissections more frequently at younger ages compared with type A. *ACTA2* mutations are also associated with PDA, which frequently recanalizes despite previous treatment.^1^

In this case, to prevent blowout rupture, urgent thoracoabdominal aortic aneurysmal replacement was considered to be most promising. However, some difficulties for proximal anastomosis via left thoracotomy were anticipated. Firstly, severe adhesion around a recanalized PDA might disrupt exposure of the proximal descending aorta. Secondly, left-to-right shunt through the PDA during the core cooling to perform open proximal anastomosis could cause systemic hypoperfusion.

## Conclusions

Stent grafting for patients with connective tissue disease is debatable owing to concerns regarding unknown effects to their fragile aortic wall.^4^ However, FET may be justified as a bridge therapy to definitive open surgery like the present staged strategy. FET is also effective for excluding residual PDA and relocating aortic trimming for distal anastomosis of TAR in patients with aortic arch disease.^2^

Whole aortic repair of a diseased lesion is advised for patients with *ACTA2* mutation because it easily dilates after dissection.^5^ Our strategy can be used to completely resect a dissected aorta while minimizing the risk and invasiveness.

## Supplementary Data

Video 1 Under lower body circulatory arrest with moderate hypothermia, patent ductus arteriosus (PDA) was sutured inside the pulmonary artery and covered with frozen elephant trunk (FET) inside the aorta to complete total arch replacement (TAR). Video available at: https://www.jtcvs.org/article/S2666-2507(23)00127-X/fulltext.

Video 2 In thoracoabdominal aortic aneurysm replacement (TAAAR), the thrombosed false lumen around the frozen elephant trunk was observed, which was used as the proximal anastomosis site. The 11th intercostal artery was reconstructed by graft interposition technique, followed by the distal anastomosis between the upper left and lower right renal arteries. Video available at: https://www.jtcvs.org/article/S2666-2507(23)00127-X/fulltext.
