# Supplementary figures and images for: Staged subtotal aortic replacement for an extensive aortic dissecting aneurysm in a 13-year-old girl with patent ductus arteriosus
Source: JTCVS Tech. 2023 Apr 19;19:22–5. doi: 10.1016/j.xjtc.2023.04.004 (PMC10268503; doi:10.1016/j.xjtc.2023.04.004)

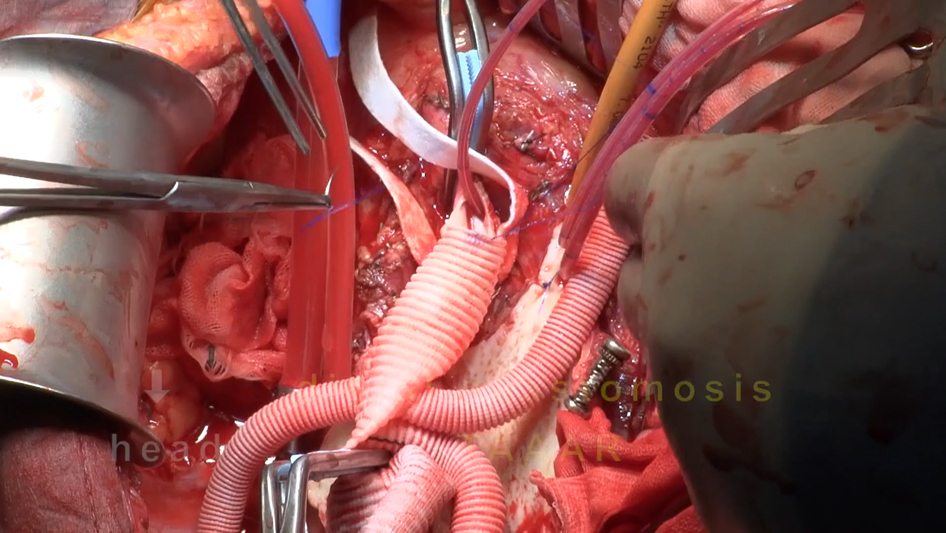

Supplement: Video 1 — Under lower body circulatory arrest with moderate hypothermia, patent ductus arteriosus (PDA) was sutured inside the pulmonary artery and covered with frozen elephant trunk (FET) inside the aorta to complete total arch replacement (TAR). Video available at: https://www.jtcvs.org/article/S2666-2507(23)00127-X/fulltext. [file fx2.jpg]

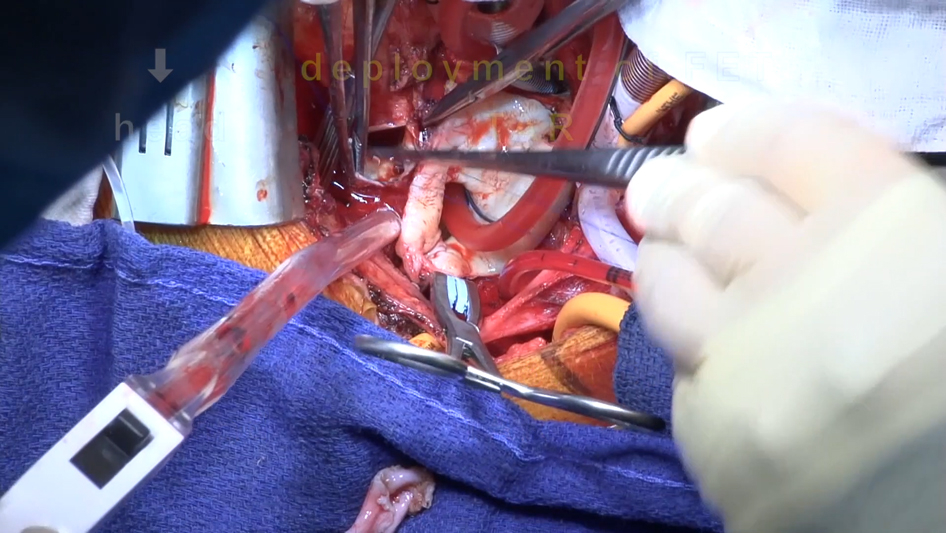

Supplement: Video 2 — In thoracoabdominal aortic aneurysm replacement (TAAAR), the thrombosed false lumen around the frozen elephant trunk was observed, which was used as the proximal anastomosis site. The 11th intercostal artery was reconstructed by graft interposition technique, followed by the distal anastomosis between the upper left and lower right renal arteries. Video available at: https://www.jtcvs.org/article/S2666-2507(23)00127-X/fulltext. [file fx3.jpg]
